# Supplementary figures and images for: A fly model establishes distinct mechanisms for synthetic CRISPR/Cas9 sex distorters
Source: PLoS Genet. 2020 Mar 13;16(3):e1008647. doi: 10.1371/journal.pgen.1008647 (PMC7108745; doi:10.1371/journal.pgen.1008647)

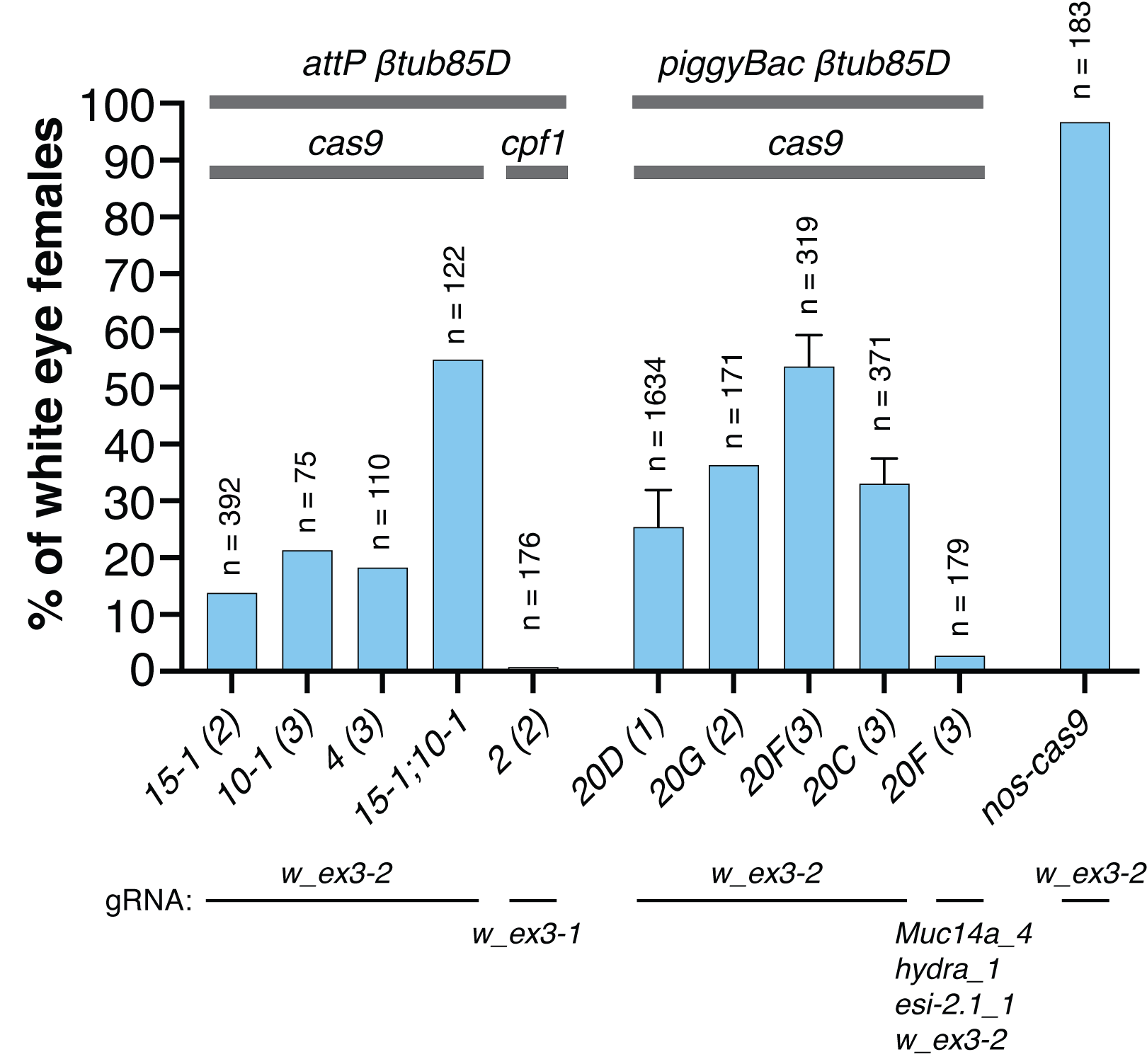

Supplement: S1 Fig — Flies carrying βtub85D-cas9 expressed from attP docking #15–1 on the second and #10–1 and #4 on the third chromosomes and from piggyBac mediated random integrations #20D on the X, #20 G on the second and #20F and #20C on the third chromosomes were crossed to lines transgenic for w_ex3-2 gRNA that targets the white gene on the X-chromosome. βtub85D-cas9/w_ex3-2 F1 males with red eyes were then crossed to white mutant females, and the female progeny scored for white eyes. Experiments combining two βtub85D-cas9 transgenes with the w_ex3-2 gRNA and βtub85D-cas9 with w_ex3-2 expressed as part of a gRNA multiplex array were also performed. Similarly, βtub85D_cpf1/w_ex3-1 red eye males were crossed to white females, and the female progeny scored for white eyes. Along with the βtub85D promoter, the nanos-cas9 transgene efficiency was tested with the same w_ex3-2 gRNA. gRNAs used for each experiment are shown below the graph. n is the number of individuals (males + females) in the F1 progeny. (TIF) [file pgen.1008647.s001.tif]

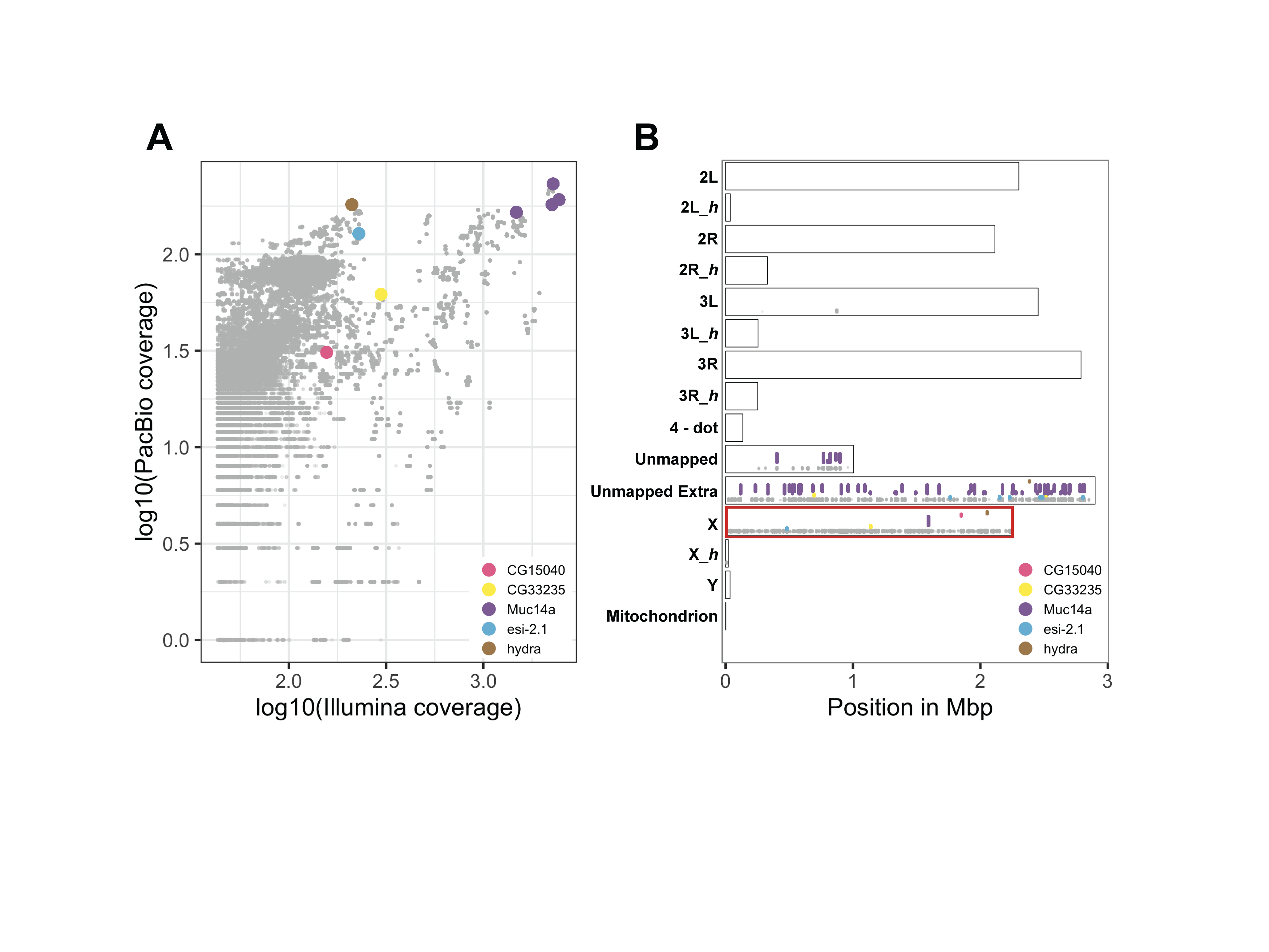

Supplement: S2 Fig — (A) Coverage of all candidate X-kmers (grey dots) including those X-kmers chosen for experimental evaluation (colored dots) within the Illumina and PacBio whole-genome sequencing read datasets as predicted by Redkmer. (B) Genomic distribution of all candidate X-kmers (grey dots) and those selected X-kmers for experimental testing (colored dots) on D. melanogaster chromosome arms based on perfect complementarity. The X-axis indicates the Mbp position of matches on each chromosomal arm, including the 4th chromosome, unmapped contigs and heterochromatin (_h). (TIF) [file pgen.1008647.s002.tif]

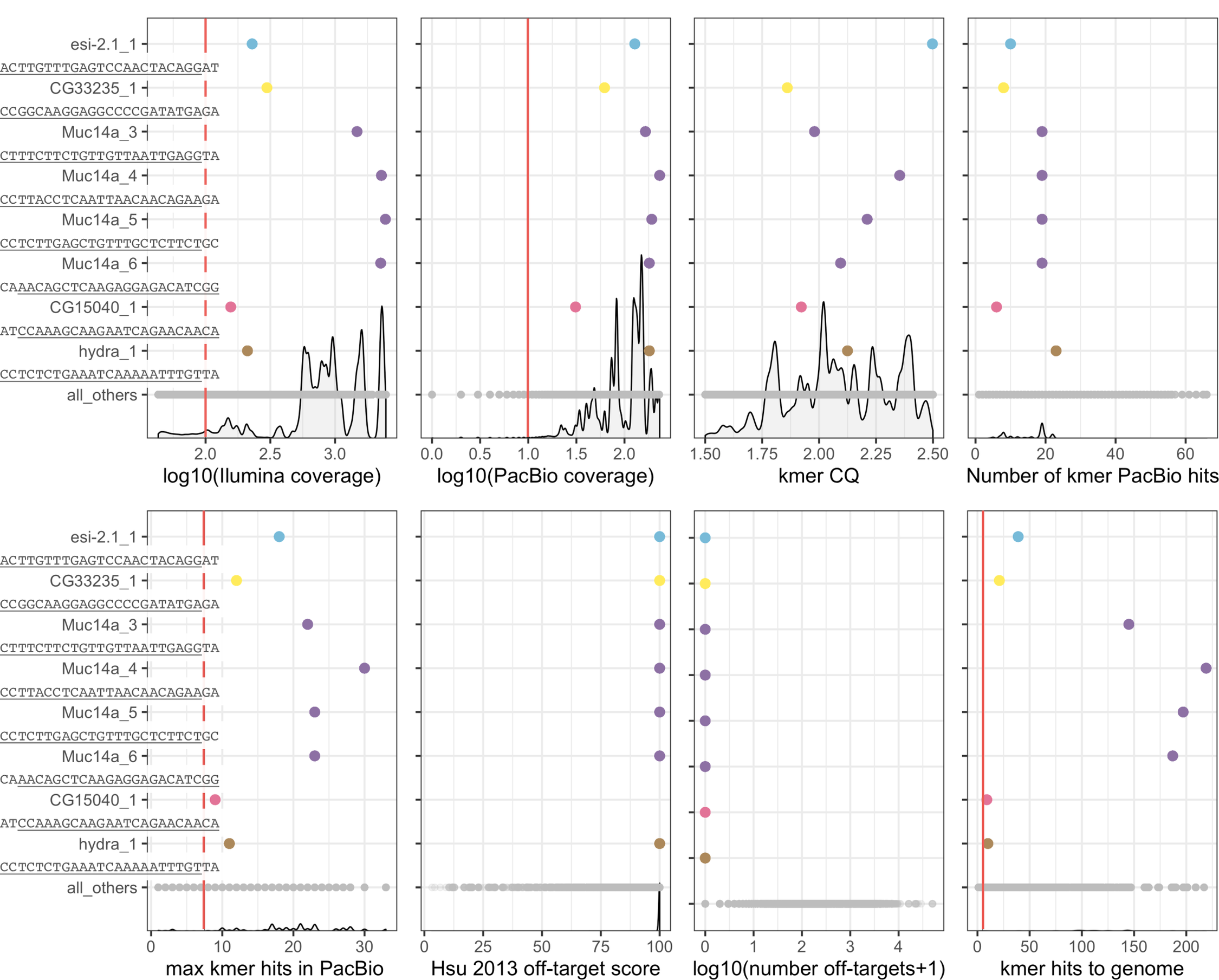

Supplement: S3 Fig — Criteria for candidate kmer selection (X-axes) are shown for each of the eight selected X-kmers for X-shredding (colored dots) and for the remaining candidate X-kmers (grey dots). Red vertical lines highlight the minimum cutoff values imposed for the final target site selection. Density plots of each criteria are also shown for the entire Redkmer candidate X-kmer output. The part of the kmer sequence that represents the target sites of experimental gRNAs is indicated (underline). (TIF) [file pgen.1008647.s003.tif]
