# Supplementary material for: A fly model establishes distinct mechanisms for synthetic CRISPR/Cas9 sex distorters
Source: PLoS Genet. 2020 Mar 13;16(3):e1008647. doi: 10.1371/journal.pgen.1008647 (PMC7108745; doi:10.1371/journal.pgen.1008647)
Supplement: Attachment — Submitted filename: Response to the reviewers.pdf [file pgen.1008647.s013.pdf]

Reviewer #1

Reviewer #1: In this study, the authors investigated mechanisms of X-shredders and "X-meddlers" in Drosophila melanogaster. The authors used their previously develop redmer software to identify suitable repeats on the X-chromosome for shredding. Using autosomal split-Cas9 and gRNA lines, they found that only one gRNA was effective at shredding, and only Cas9 with the beta tubulin promoter, not nanos, despite the latter having overall more cleavage activity. This was likely due to the difference in expression timing between these promoters. The "X-meddler" involved targeting a haplolethal gene on the X-chromosome and represents the first experimental demonstration of a genetic control system based on this strategy (though the final proposed system would need to be on the Y chromosome). This strategy was also successful in biasing the sex ratio toward males.

This manuscript represents a solid step forward in the field and should be published in PLOS Genetics. Here's some comments on how the authors could potentially improve their manuscript (in roughly the order they appear in the text):

1. The abstract is somewhat light on actual results. Maybe add 2-3 lines regarding the conclusions?

We added a few more details to the abstract.

2. In the introduction, the authors say, "This is because the fecundity of females, the sex with a lower rate of gamete production, generally determines the size of a population." While playing a bit part, I'm not sure if this is technically accurate, particularly given the high density-dependent mortality in mosquito larvae. I'd suggested softening the wording of this phrase.

This statement was weakened and now reads "One factor is the fecundity of females, the sex with a lower rate of gamete production, which can exert a large influence on the size of a population."

3. At the end of the first paragraph of the introduction, the authors may want to clarify that the experimental systems have the X-shredder allele on the autosome, so while a big step towards Driving Y elements, they don't have the power of the Driving Y/X-shredder described in the middle of the paragraph. Additionally, only one of these experimental systems was used in a cage study.

A sentence was added as suggested.

4. It would be nice to have a good term for the X-linked haplolethal targeting strategy, but "X-meddling" is not very informative with regard to the goals of the meddling. I'd encourage the authors to reconsider the name. Maybe "X-poisoning" would be good? Perhaps "X-disrupting" if you want to be less flamboyant?

We spent a long time going back and forth between terms, X-poisoning was also on the list and we are ok reverting to it.

5. a. For the second paragraph in the results, it may be helpful to mention the specific range of the rate of white-eye offspring for the βtub85D promoter, to make it clear why nanos works better here.

This info was added.

b. Also "LbCfp1" should be "LbCpf1" in one spot. In general, be careful with your cpf1/Cas12a/LbCpf1 terminology.

This was fixed.

c. Also, this giant paragraph should probably be broken into smaller more focused paragraphs based on the white results, target determination, and results of the new constructs. I'd suggest adding subsection headings to the results as well.

Agreed, subheadings were now added.

d. "the RpS5a (McKim, Dahmus, and Hawley 1996), RpS6 (Stewart and Denell 1993) ribosomal protein genes"

should be “the RpS5a (McKim, Dahmus, and Hawley 1996) and RpS6 (Stewart and Denell 1993) ribosomal protein genes”.

This was rectified.

e. Was the ability of Muc14a_1 and Muc14a_2 to disrupt the target confirmed, as in figure 2A?

These gRNAs hit in a less repetitive 5’ region of the gene which is not on the amplified fragment used in figure 2A. We did independently confirm activity in vitro for the Muc14a_1 gRNA:

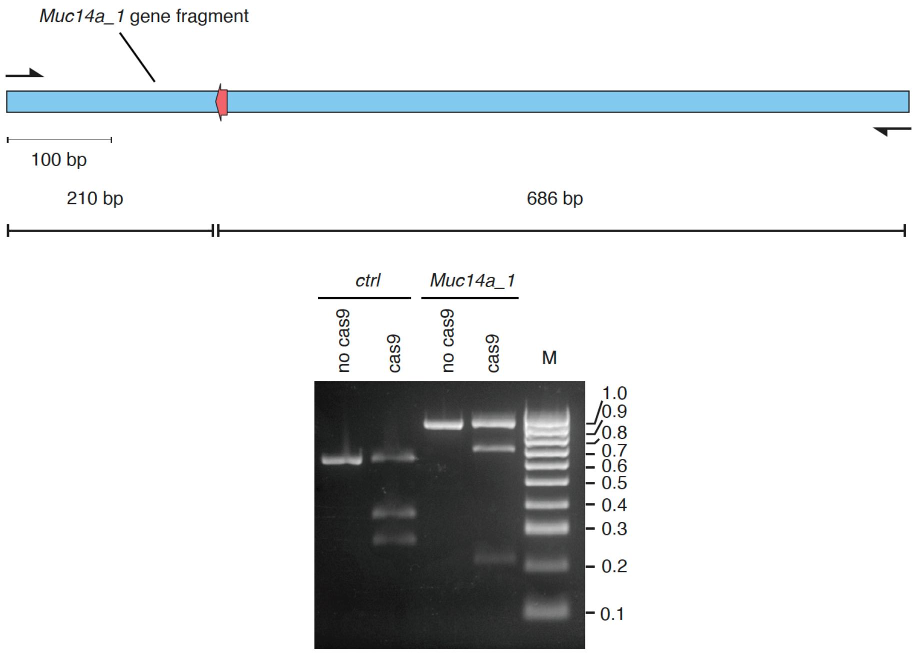

6. a. In the third paragraph of results, the authors use the phrase “the ubiquitous Pol III promoter of the Drosophila U6 snRNA gene”, but I think they mean “the Pol III promoter of the Drosophila U6 snRNA gene that drives ubiquitous expression”.

This was fixed.

b. The author write, “We also observed that the w_ex3_2 gRNA targeting white, when encoded as the ultimate of four gRNAs within the array, showed a dramatically reduced level of activity (Figure 2B).” This should be Figure S1. This is likely due to saturation of the Cas9 activity by the gRNAs (this study covers multiplex gRNAs and Cas0 saturation and should be published in Science Advances soon: https://www.biorxiv.org/content/10.1101/679902v1), likely combined with reduced expression of gRNAs in time for cutting by last gRNA due to the tRNA system.

This consideration plus citation was added (in the discussion).

c. Could the term “meiotic activity” be clarified, especially with respect to the usual expression pattern of the two promoters that are compared in the manuscript? For example, is it after meiosis I, or during all of meiosis?

We modified the sentence summarizing what is known expression pattern of the tubulin promoter. It now reads “at the primary spermatocyte stage (at the onset of Meiosis I)”.

7. a. Statistics are missing on Figures 2C and 4C for the % males and % females (it’s pretty clear, but probably good to add for completeness).
b. Also, it may be better to eliminate the “%” from the category labels. It’s already on the vertical axis, and it could create some confusion in some cases if people go through the figures too quickly (eg, “why don’t the 40% males and 5% females add up to 100% in the brown part of Figure 4C).
c. Also, the legend in 2C has “blue grey white”, but the figure itself has “white grey blue”.

d. There is some redundancy in this figure that could potentially distort useful information. Instead of the current form, it might be better to use egg to pupae survival rates and pupae to adult survival rates instead of absolute % of eggs, and then just have a single column for sex ratio. The sample size could go into a supplement, clearing up the figure quite a bit and putting more focus on what is happening at each stage, rather than having to keep overall trends in mind.

We rearranged the data in the figures as suggested and corrected the labels. Statistics were added.

e. Any idea why the βtub85D-Cas9 line had lower hatch rates, especially in 2C?

We have been seeing some variation when it comes to the toxicity/cost of expressing Cas9, which is why we always compare only within each experiment i.e. between the Cas9 line and the added effect given by the gRNA with the same Cas9 insertion with flies reared parallel alongside each other. It may be related to temperature, and occurs in different insertions/strains, but we don't know much. Cas9 toxicity has been described previously and anecdotally Cas9 seems often to have a higher fitness cost in Drosophila as compared to Anopheles mosquitoes.

8. For Figure 4D, only the "A" in "ACC" and the "AT" in "ATG" are highlighted green. However, perhaps all three nucleotides should be highlighted in both cases, representing insertions where some of the nucleotides just happen to be similar to other parts of the sequence?

This was fixed.

9. Were the RpS5a and RpS6 targets tried with the nanos-Cas9 line? Since nanos seems to have higher activity, this might substantially increase efficiency. It would be cool to see a little bit of data for this and might be easy to do, but I don't consider it necessary if the authors don't have time before submitting their revision.

A fair point. We have now done this experiment since it appears that this result is of particular interest to others (I also received emails about this). The result was complete male sterility. We believe that this simply reflects the fact that ribosomal gene function is required during spermatogenesis and knocking out X-linked ribosomal genes already in male stem cells simply cannot work. We now present the result in the text and the raw data are found in Supplementary Dataset 1. We also added above considerations to the text.

10. I may have missed this, but could be better gRNAs simply have intrinsically more activity, rather than needed to invoke sequence microenvironments or other explanations? If this is true, it could possibly go against the conclusion that a single gRNA is best. Perhaps a cluster of different gRNAs that each are very active could still be superior, but if a superior gRNA is put with a cluster of inferior ones, it gets too "diluted" and total cleavage is reduced.

Entirely possible, our conclusion was more based on what can be practically achieved. Finding the one gRNA that works well is already a challenge. We do now also mention intrinsic activity as a factor.

11. It looks like the repeats fall within a narrow range, rather than scattered throughout the X-chromosome. Is this different than the Anopheles experiments? It might be worth commenting that targeting a narrow chromosomal window may actually improve the chance for successful repair (since the little pieces that form won't matter - only the big pieces need to be rejoined. If targets were scattered throughout, then each piece may need to be properly repaired, or at least lots of them). This may not have been seen when multiplexing gRNAs in the current study since only a limited number of gRNAs seemed to be highly active.

The mosquito rDNA is also just a single cluster, one very long array of repeat units (500–700 repeats per genome) with each repeat (and hence the distance between X-shredding cut sites) about 9 kb long. In Drosophila there are 271 base pairs between Muc14a_6 repeats and short distances also for the other targets and this shorter distance could indeed have increased the chances for repair. This consideration was added to the discussion.

There does not appear to be a high-confidence high-copy distributed X-linked repeat in either species. Such a sequence may exist in other species and the effect on X-shredding remains to be explored.

12. Comma after "Chromosome-wide, distributed repeats represent an alternative set of targets".

We fixed this.

13. "Alternatively, the observed differences could also partly relate to the predominance of homologous over non-homologous DNA repair pathways acting with varying stringency during the early (including stem cells) and late stages of spermatogenesis, respectively (Chan et al. 2011)." Why would homology-directed repair impact X-shredding when no template for repair is available in males? Perhaps a simple explanation of nanos vs βtub85D X-shredding efficiency is that despite the greater overall activity of nanos, repair is highly efficient in pre/early meiosis and less so in late meiosis when βtub85D has higher activity?

With repetitive sequences homologous repair could operate between repeats. For example, gene conversion is the mechanism proposed to be responsible for the homogenization of multigene families such as the nuclear ribosomal gene clusters. We barely understand what happens within repeats e.g. how elements (e.g. R1 and R2 retroelements) are counterbalanced by homologous repair. In short, we did not want to rule out homologous repair here even if no homologous chromosome is present.

We are now also mentioning the possibility that repair may simply be more efficient early on.

14. "The fly model of X-shredding we have established will allow to tackle these questions experimentally" should instead be something like this: "The fly model of X-shredding we have established will allow these questions to be tackled experimentally".

This was corrected.

15. Maybe comment that X-meddling may be easier to perform from the Y chromosome due to the need for reduced Cas9 activity (just one or a few cuts needed vs. potentially dozens or hundreds). Also, conserved sites wouldn't necessarily be needed if multiplexed gRNAs were used, as long as they all had good activity.

These considerations were added to the discussion.

16. Regarding the possibility of dominant negative mutants in the RpS genes, I think this may certainly be the case. However, could another possibility simply be that the gene isn't completely haplolethal, and that after passing a certain "critical" stage, likely in embryo development, the gene is haplosufficient? This may be a boring explanation in comparison, but it could explain why you detected some frameshift mutations in viable individuals. This could be assessed by crossing these females (presumably before sequencing) and observing if all the male progeny are viable (in which case, likely dominant negative) or if most are still nonviable (then likely not completely haplolethal). This could be left to a future study.

These considerations were added to the discussion.

Minor note: For future submissions, I'd encourage the authors to put the figures and their legends "in-line" with the text if the journal allows submissions like this (I received the same advice when submitting a PLOS Genetics article a while ago, and so far, all journals I've worked with seem to allow this now). Bigger spaces between paragraphs would also make the manuscript easier to read.

Overall, good job by the authors. This manuscript should fit in well at PLOS Genetics. I am Jackson Champer, and I'm happy to clarify any of the above points if my wording doesn't make sense (jc3248@cornell.edu).

Reviewer 2

Reviewer #2: This manuscript explores the general hypothesis that interfering with X chromosomes during spermatogenesis can impact the sex ratio of progeny derived from affected males. Such a mechanisms, known as X-shredding, has been show to be successful in the malaria mosquito, Anopheles gambiae, and has been proposed as a method for population suppression. However, aside from rare naturally occurring examples of biased sex chromosome transmission, little has been done in Drosophila to look at such systems - given the recent rise in the population of D. suzukii and other dipteran pests, a better understanding of potential genetic control strategies is welcome.

Here the work describes the application of two CRISPR-Cas9 based approaches; X-shredding, where X-specific repeats are targeted for cleavage in the male germline, and X-meddling, a proposed method for sex-biasing populations by targeting halo-insufficient loci such that the homogametic progeny die. Overall, the experiments are well designed and demonstrate that both methods can be applied in Drosophila, suggesting a broader applicability of the approaches, at least in diptera. Key new insights are the use of computational approaches to identify X-specific repeated DNA sequences and showing that at least in one situation targeting such a sequence can bias the sex ratio. Second, the experimental verification of the hypothesis of Burt and Deredec that targeting an X-linked haploinsufficient locus in the male germline could reduce the viability of XX progeny.

While overall the work is interesting and supports the further research into these proposed control mechanisms in a tractable system, I feel the current manuscript leaves much hanging. In particular, there is minimal discussion of, or investigation into, why only one of the Muc14a gRNAs is sucessfull in vivo; aspects of the mutation spectrum generated with the early nos-Cas9 driver versus the later B-tub-Cas9 are poorly explained, in particular the expected outcomes when repair is via HDR or NHEJ is poorly explored.

There isn't much data out there looking at expected outcomes when a repeat sequence is targeted by endonucleases in the germline. We are not aware of examples in the literature. We also were very careful not to over-claim on our findings or what our results can show. For example, the reduction of the repeat size from 100 to 5 repeats (by NHEJ and loss of fragments) may or may not look very similar to the conversion of half of the repeats in the cluster from one type to another (by HDR with no reduction in the number of repeats) when we look at this by amplicon sequencing. There is a danger of reaching false conclusions. Delving into this further will require further work in the future and we agree with the reviewer that much remains to be done.

I note that the use of the attached X stock to isolate cut chromosomes in the male is elegant, and the assays in repair pathway mutants are good. I found the discussion around Fig 4 patchy and lacking firm conclusions, while targeting two X-linked ribosomal protein genes did, as predicted, induce male biased progeny, the mechanism for this remains unclear and I would have expected more than just a suggestion it is a dominant effect.

We tried to improve the discussion of these aspects in this revision. However, we don't believe any new mechanism needs to be invoked necessarily. When we observe female lethality after a modification of their fathers X-linked ribosomal gene copy only two parsimonious hypotheses come to our mind (and both are already present in the literature which we cited):
Either expression from only one copy of such a ribosomal gene is insufficient for female viability or the modified copy inherited from the father itself induces the lethal effect. While we started working under the first hypothesis our results started to point more towards the second (female survivors apparently not inheriting a functional parental copy).

In places the manuscript appears hastily prepared and lacks clarity, particularly in the early sections describing the generation and assay of the Cas9 lines.

We tried to improve the manuscript throughout.

Minor comments:

Pg3 & Fig S1- lack of clarity on the Cas9 lines used in the white assays.
FigS1 Legend is wrong #20G on 2nd and #20F and #20G on 3rd

This was fixed and the description hopefully improved.

Pg3 & FigS2 & S3 - Lacks clarity on Redkmer selection - matches to Unmapped and unmapped extra reads should be properly explained.

See below, response to reviewer 3.
Briefly, matches to the unmapped and unmapped extra contigs are likely hits to parts of the X chromosome that are repetitive and as a result could not be placed correctly in the official assembly.

Pg4 & Fig1 Results with esi2 seems odd, generating female bias, and is not commented upon

It's the female control that gives a female bias for esi2. Generally, there is no Cas9 expression expected in females, also note that here the total numbers counted are not high (328), compared to some of the other experiments (e.g. 3904 for Muc14a_6). We did not follow up on whether this is a true effect as we were trying to find a convincing and reproducible example of a gRNA giving a male bias phenotype which is more interesting from the perspective of application.

Pg4 why is there is no female control for Muc14a_6

We used either female or no gRNA control in parallel to every cross throughout our experiments depending on what was easier to generate/at hand.

We did at some point perform female controls in multiple crosses for different *βtub85D-cas9* lines with the Muc14a_6 gRNA and the number of females and males were the same (47.8% females vs 52.2% males for line #4 and 53.9% females vs 46.1% males for line #10-1. These were done with Cas9 lines on the 3rd chromosome:

The F1 progeny from the crosses between *βtub85D_cas9/sgRNA* and wild type *w* lines				
**female X male**	**females**	**males**	**females %**	**males %**
w X ***Muc14a_6**/βtub85D-cas9*^{4}	182	265	40.7	59.3
***Muc14a_6**/βtub85D-cas9*^{4} X *w*	178	194	47.8	52.2
w X ***Muc14a_6**/βtub85D-cas9*$^{10-1}$	78	118	39.8	60.2
***Muc14a_6**/βtub85D-cas9*$^{10-1}$ X *w*	319	273	53.9	46.1

Pg5 data on w with gRNA array is in fig S1not Fig 2

This was corrected.

Pg5 Comment on the lethality associated with the Cas9 alone

See above reviewer 1.
Briefly, we something see Cas9 toxicity, we are always comparing only within each experiment (cas9 vs cas9+gRNA).

Pg5 & Fig2C, male bias looks much less than in 2B?

In figure 2C, the male rate had been calculated on the eggs and not as a fraction of the adults as in figure 2B. This figure had a mistake, was confusing, and we have rearranged the data in the figure as suggested by reviewer 2 (see above).

Pg6 & Fig 3B - why less replicates with Control & nos-Cas9

For the control we assumed this would show us the repeat structure before modification and indeed ~90% of reads contain the target site. There are differences between individuals and more wild type samples would undoubtably have unearthed more variation in the Muc14a cluster but we also have various Pacbio reads that span this repeat and also the genome assemblies to study the wild type structure so we didn't think we would learn that much. For the *nos* samples I believe this is what we had available at the time, although more is always better.

Pg6 & Fig 3C - helpful if changes from wild type allele were highlighted

Indeed, differences are now indicated in green.

Reviewer 3

Reviewer #3: In this paper, Fasulo and colleagues create experimental synthetic sex ratio distorters in Drosophila melanogaster. "X-shredding" and "X-meddling" are two distinct strategies that were confounded in previous experiments. The experiments in this paper demonstrate that "X-shredding" can work outside of mosquito and therefore could be promising for uses beyond malaria control.

I think that this paper is broadly interesting. I like that the authors report on differences in timing (nos versus Btub85D) and the induced mutations, as these experiments give some insight into the underlying repair processes. The methodology in the paper is straightforward. Below I make some suggestions that I hope will improve the manuscript.

Major comments:
I do not see any tables except for Table S1. I only see reference to Tables 1 and S1, 3 and 5 in the manuscript.

We now uploaded "Supplementary Dataset 1" that contains all genetic crossing data for all experiments.

Figure 3 legend: "percentage of all mapping reads" is confusing. Do you mean the percentage of all mapped reads? These data should appear somewhere.

This was corrected.
As for the data, it depends what is meant here, we are certainly willing to upload the raw fastq data to SRA or ENA. Alternatively, the allele frequency tables after mapping for all samples can be found here: (and a link to this could be included) https://github.com/genome-traffic/flyXpaper

In panel B, why do you think that the control only has 90%? Why would nos-cas9 have fewer unique alleles lacking full gRNA target site than the control?

As mentioned, there is pre-existing repeat heterogeneity in the cluster in wild type individuals (Figure 3C shows some variants of the target site). As for nos-cas9 having even fewer unique alleles, this is possibly because the cluster has shrunk in total repeat size due to continued cleavage and repair cycles from stem cells onwards. If for example only 5 repeat units are left, we would have reduced the number of unique alleles to a maximum of 5 and a minimum of 1.

Figures 2C and 4C: I think that you need a better description of the categories (e.g. % male and % female) in the legend. The % hatching is lower for Btub85-cas9/+ (Fig 2C). Is this chromosome different from the Btub85-cas920F used in X-meddler experiments?

We have changed the way we present that data and fixed some mistakes along the way.

Figure 3A: I like the schematic showing the PCR and amplicon sequencing but I had a few questions. Are the thin arrows the primers? And does the 153 bp region correspond to the repeat unit? What are the grey bars flanking the blue bar?

Correct, the thin arrows on the repeats are primers, the 153 bp corresponds to the repeat unit and the grey boxes are Illumina adapters. This is now better explained in the legend.

Ideally, the repeats targeted for X shredding would be X-specific for future applications, to reduce the chance of off target effects that may be transmitted. Although your sex ratio data show that the X chromosome is likely targeted, it would be good to give the reader an idea of how enriched these repeats are on the X chromosome. In Figure S2B, you show that the Muc14a target sites are frequent on 'Unmapped_Extra' and 'Unmapped' and so their locations are unknown. It may not be possible to figure out where all of these repeats are with current technology, but there are at least three things that you could do to add support for the majority of these repeats being X-linked: 1) FISH on polytene and mitotic chromosomes, 2) map to an assembly that has better representation of heterochromatin (https://doi.org/10.1534/genetics.118.301765), 3) plot Illumina depth in male vs female.

The sequences we have targeted are specific to the *D. melanogaster* X chromosome, as far as this can be said with certainty. The 'Unmapped_Extra' and 'Unmapped' are collections of unmapped scaffolds in the

latest, official D. melanogaster assembly. The presence of hits within these collection does not indicate necessarily that these are hits to other chromosomes. Rather, because these are repeats, they could not be placed correctly in the final (X) assembly.

We now checked our kmers against the improved assembly from Chang el al (2019, Genetics) that the reviewer suggested (which wasn't available at the time). The figure below shows that all target sites used in this study had hits exclusively within one scaffold (X1) that is located on the X-chromosome. We mention this now in the paper (we show the data in Table S2) and cite the new assembly as an additional control.

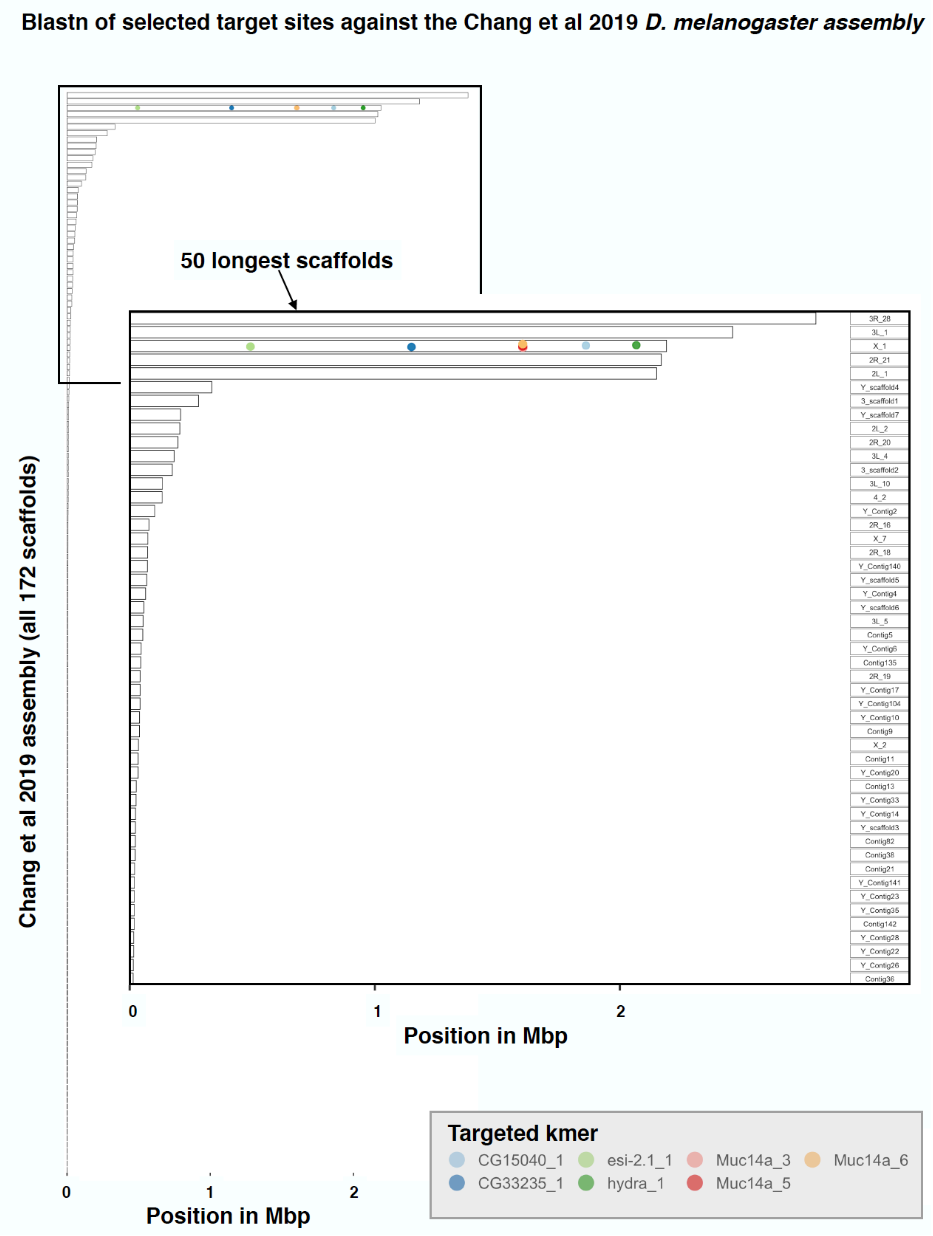

It is important to note that for virtually all agriculturally or medically important target species such high-quality X assemblies will not be at hand. This is why the redkmer pipeline was designed to only rely on raw sequence input. Our previous paper describes this bioinformatic pipeline and we discussed in detail and provided evidence for the X-specificity of redkmer target kmers. This additional control lends further support to this.

Regarding the reviewer's final point (Illumina depth in male vs female), this can be found in the Figure S3 (panel showing kmer CQ – i.e. the ratio of reads in females vs males) where all target sites used display a CQ ~ 2, indicating X-chromosome linkage.

The proof-of-principle experiments described in this paper are a necessary and important step towards developing tools intended for vector control. However, I think that it is appropriate to add a sentence or two that mentions some of the caveats for using an X shredder designed to target X-linked repeats. For example, targeting an X-enriched repeat that is dispersed in the genome, or even dispersed on the chromosome, may cause genome rearrangements of unknown consequence that can be transmitted. I think that you need to know more about the targets and their distribution in the genome (and repair processes involving repeats).

We added a sentence with these considerations.

Minor:
Figure 2: The figure was not completely clear to me ("a typical region of the Muc14a gene…") until I saw Figure 3. It might help to add more labels, or more description to the legend. Alternatively, combining with the schematic in Figure 3 could help.

This was indeed confusing; we changed the wording and improved the description. The fragment we amplified, cloned, sequenced and used for the in-vitro digest actually contains more than one repeat unit. We did this to ensure that the target sequence of all 4 Muc14a gRNAs that target the cluster were present. We also improved references to figure 3 in the text.

Page 3: About the Drosophila X-linked repeats, it's not clear what you are comparing to when you say that they are less abundant.

This was made clearer.

Page 4: It would help to describe the target repeats in a bit more detail here (e.g. repeat unit, distribution, composition).

We have tried to further improve the description of the Muc14a repeat.

Pages 4 and 5: Cytology on sperm (e.g. FISH with X-satellite probe) may help you determine if these sperm are lost.

We established the first X-shredding system in Anopheles already in 2008 with a couple of follow up papers. We performed may cytological experiments including FISH experiments. However ultimately, they proved inconclusive, nothing was ever published. This was actually one reason we eventually decided we needed to establish a genetic more tractable system to understand what was going on. I agree that *Drosophila* offers more power and we could attempt this again.

Pg 5, line 4 "the ultimate of four gRNAs" is unclear.

This was explained better.

Page 6 lines 1-4: The wording here is hard to follow.

Agreed, this was reformulated.

Have all data underlying the figures and results presented in the manuscript been provided?
Large-scale datasets should be made available via a public repository as described in the *PLOS Genetics* data availability policy, and numerical data that underlies graphs or summary statistics should be provided in spreadsheet form as supporting information.

Reviewer #1: Yes

Reviewer #2: Yes

Reviewer #3: No: I could not find any tables except Table S1

We now included all raw data as Supplementary Dataset 1.

PLOS authors have the option to publish the peer review history of their article (what does this mean?). If published, this will include your full peer review and any attached files.

If you choose “no”, your identity will remain anonymous but your review may still be made public.

Do you want your identity to be public for this peer review? For information about this choice, including consent withdrawal, please see our Privacy Policy.

Reviewer #1: No

Reviewer #2: No

Reviewer #3: No
